# Role of SpoIVA ATPase Motifs during Clostridioides difficile Sporulation

**DOI:** 10.1128/JB.00387-20

**Published:** 2020-10-08

**Authors:** Hector Benito de la Puebla, David Giacalone, Alexei Cooper, Aimee Shen

**Affiliations:** a Department of Molecular Biology and Microbiology, Tufts University School of Medicine, Boston, Massachusetts, USA; b Graduate Program in Molecular Biology and Microbiology, Tufts University School of Medicine, Boston, Massachusetts, USA; c Department of Microbiology and Molecular Genetics, University of Vermont, Burlington, Vermont, USA; Ohio State University

**Keywords:** ATPase, *Clostridioides difficile*, SipL, SpoIVA, spore coat, sporulation

## Abstract

The nosocomial pathogen Clostridioides difficile is a spore-forming obligate anaerobe that depends on its aerotolerant spore form to transmit infections. Functional spore formation depends on the assembly of a proteinaceous layer known as the coat around the developing spore. In C. difficile, coat assembly depends on the conserved spore protein SpoIVA and the clostridial-organism-specific spore protein SipL, which directly interact. Mutations that disrupt their interaction cause the coat to mislocalize and impair spore formation. In Bacillus subtilis, SpoIVA is an ATPase that uses ATP hydrolysis to drive its polymerization around the forespore. Loss of SpoIVA ATPase activity impairs B. subtilis SpoIVA encasement of the forespore and activates a quality control mechanism that eliminates these defective cells. Since this mechanism is lacking in C. difficile, we tested whether mutations in the C. difficile SpoIVA ATPase motifs impact functional spore formation. Disrupting C. difficile SpoIVA ATPase motifs resulted in phenotypes that were typically >10^4^-fold less severe than the equivalent mutations in B. subtilis. Interestingly, mutation of ATPase motif residues predicted to abrogate SpoIVA binding to ATP decreased the SpoIVA-SipL interaction, whereas mutation of ATPase motif residues predicted to disrupt ATP hydrolysis but maintain ATP binding enhanced the SpoIVA-SipL interaction. When a *sipL* mutation known to reduce binding to SpoIVA was combined with a *spoIVA* mutation predicted to prevent SpoIVA binding to ATP, spore formation was severely exacerbated. Since this phenotype is allele specific, our data imply that SipL recognizes the ATP-bound form of SpoIVA and highlight the importance of this interaction for functional C. difficile spore formation.

**IMPORTANCE** The major pathogen Clostridioides difficile depends on its spore form to transmit disease. However, the mechanism by which C. difficile assembles spores remains poorly characterized. We previously showed that binding between the spore morphogenetic proteins SpoIVA and SipL regulates assembly of the protective coat layer around the forespore. In this study, we determined that mutations in the C. difficile SpoIVA ATPase motifs result in relatively minor defects in spore formation, in contrast with Bacillus subtilis. Nevertheless, our data suggest that SipL preferentially recognizes the ATP-bound form of SpoIVA and identify a specific residue in the SipL C-terminal LysM domain that is critical for recognizing the ATP-bound form of SpoIVA. These findings advance our understanding of how SpoIVA-SipL interactions regulate C. difficile spore assembly.

## Abstract (precis)

The major pathogen Clostridioides difficile depends on its spore form to transmit disease. However, the mechanism by which C. difficile assembles spores remains poorly characterized. We previously showed that binding between the spore morphogenetic proteins SpoIVA and SipL regulates assembly of the protective coat layer around the forespore. In this study, we determined that mutations in the C. difficile SpoIVA ATPase motifs result in relatively minor defects in spore formation, in contrast with Bacillus subtilis. Nevertheless, our data suggest that SipL preferentially recognizes the ATP-bound form of SpoIVA and identify a specific residue in the SipL C-terminal LysM domain that is critical for recognizing the ATP-bound form of SpoIVA. These findings advance our understanding of how SpoIVA-SipL interactions regulate C. difficile spore assembly.

## INTRODUCTION

The Gram-positive spore-forming bacterium Clostridioides difficile, formerly known as Clostridium difficile, is a leading cause of health care-associated infections worldwide (1). C. difficile causes a debilitating diarrhea that can lead to severe complications like pseudomembranous colitis and toxic megacolon (2). These disease symptoms are caused by the glucosylating toxins produced by C. difficile (3), although disease typically occurs only in individuals experiencing gut dysbiosis, because dysbiosis is required for C. difficile to establish a replicative niche in the gut (4, 5). While C. difficile is intrinsically resistant to many antibiotics, its metabolically dormant spore form further facilitates C. difficile survival because spores are inert to antibiotics and can persist in the environment for long periods of time (6).

Spores are also the major transmissive form of C. difficile, since it is an obligate anaerobe (7, 8). As a result, spore formation is critical for C. difficile to survive exit from the host and persist in the environment (9). This important developmental process involves a series of coordinated morphological changes that begins with the formation of a polar septum, which creates a larger mother cell and smaller forespore (10, 11). The mother cell then engulfs the forespore, suspending the double-membraned forespore within the cytosol of the mother cell. Following engulfment, a thick layer of modified peptidoglycan known as the cortex forms between the two membranes of the forespore. The cortex layer is critical for maintaining metabolic dormancy and confers resistance to heat and ethanol (12). As the forespore develops, a series of proteins localizes to and assembles on the outer forespore membrane to form concentric proteinaceous shells around the forespore known as the coat, which protects spores against enzymatic and chemical insults like lysozyme and quaternary amines (13).

The molecular mechanisms controlling these developmental stages have been extensively characterized in Bacillus subtilis, and factors critical for each of these morphological stages have been identified (10). While most of these B. subtilis morphogenetic factors are conserved across spore formers (14, 15), analyses of spore assembly in C. difficile indicate that many of these factors have different functions or requirements in C. difficile. The conserved transmembrane protein SpoIIM is essential for engulfment in B. subtilis (16) but dispensable for this process in C. difficile (17, 18), while the lytic transglycosylase SpoIID is essential for engulfment in B. subtilis (19) but only partially required in C. difficile (17, 18). Although components of a channel complex are required to maintain the integrity of the forespore in both B. subtilis and C. difficile (20–22), this complex is required for engulfment in C. difficile (21, 22) and yet dispensable under some conditions in B. subtilis (23).

In addition, C. difficile and B. subtilis differ considerably in how they assemble the coat layer since only two of the nine coat morphogenetic proteins identified in B. subtilis have homologs in C. difficile, namely, SpoVM and SpoIVA (24). While both these proteins are conserved across spore formers (14, 15), they are differentially required in C. difficile relative to B. subtilis because SpoVM is largely dispensable for functional spore formation in C. difficile (25), in contrast with B. subtilis (26). In B. subtilis, SpoIVA and SpoVM are the first proteins to be recruited to the outer forespore membrane during engulfment, and both are essential for coat and cortex assembly and thus functional spore formation (26–30). SpoVM recognizes the positive curvature of the forespore and embeds itself in this membrane (28, 31). SpoVM directly recruits SpoIVA to the forespore (32) and potentiates the polymerization of SpoIVA around the forespore (33). SpoIVA is a cytoskeletal-protein-like protein with ATPase activity that encases the forespore in an ATP-dependent manner (34, 35) when bound to SpoVM (32). SpoIVA subsequently recruits additional coat morphogenetic proteins (27, 36). Thus, these two proteins form the basement layer on which the coat assembles in B. subtilis.

While C. difficile SpoIVA also regulates coat assembly and is essential for functional spore formation, C. difficile *spoIVA* mutants produce a cortex (37), unlike their counterparts in B. subtilis (27). Furthermore, C. difficile *spoVM* mutants exhibit only an ∼3-fold decrease in functional, heat-resistant spore formation (25) compared with the ∼10^6^-fold defect observed in B. subtilis *spoVM* mutants (26). The relatively minor defect of C. difficile *spoVM* mutant spores is likely because C. difficile does not carry a quality control pathway that eliminates defective sporulating cells in B. subtilis (29, 38).

This quality control mechanism senses defects in B. subtilis SpoIVA localization around the forespore, which can occur due to a loss of SpoVM or SpoIVA ATPase activity (29). Notably, the quality control pathway only appears to be conserved in spore formers of the order *Bacillales* (39), suggesting that clostridial organisms either lack a mechanism for eliminating spores with coat assembly defects or employ a clostridial-organism-specific quality control mechanism.

Another major difference in coat assembly between C. difficile and B. subtilis is the requirement for SipL, a SpoIVA-interacting coat morphogenetic protein that is only conserved in the *Clostridia* (37). C. difficile *sipL* mutants phenocopy C. difficile *spoIVA* mutants in that they exhibit similar defects in coat localization and heat-resistant spore formation despite making a cortex layer (37). We previously showed that SipL directly binds SpoIVA through the SipL C-terminal LysM domain (37) and that disrupting SipL LysM domain binding to SpoIVA via mutation of specific residues causes defects in coat assembly and heat-resistant spore formation (40).

While SipL residues important for recognizing SpoIVA have been identified, the SpoIVA residues recognized by the SipL LysM domain remain unknown. We previously showed that mutating a residue predicted to prevent C. difficile SpoIVA from binding ATP also reduced SpoIVA binding to SipL in coaffinity purification assays performed on Escherichia coli lysates (37). Since previous work with B. subtilis SpoIVA revealed that ATP binding to SpoIVA induces a conformational change that allows SpoIVA to self-polymerize upon hydrolyzing ATP (34, 41), we sought to test whether ATP binding and/or hydrolysis were required for C. difficile SipL to recognize SpoIVA during spore formation. Since the ATPase activity of B. subtilis SpoIVA is also required for SpoIVA to fully encase the forespore (34), we wondered how disrupting conserved ATPase motifs in C. difficile SpoIVA would impact spore coat assembly. To this end, we mutated C. difficile the SpoIVA conserved ATPase motifs and determined their impact on C. difficile spore formation, binding to SipL, and coat localization. Our analyses indicate that SipL preferentially recognizes the ATP-bound form of SpoIVA and reveal further differences in the functional requirements for conserved spore proteins between B. subtilis and C. difficile.

## RESULTS

### Alanine mutations in SpoIVA ATPase motifs do not strongly reduce C. difficile heat-resistant spore formation.

The N terminus of SpoIVA has homology to the translation factor (TRAFAC) clade of GTPases (see Fig. S1 in the supplemental material) (34), since SpoIVA carries three of four highly conserved motifs that distinguish TRAFAC GTPases (and the larger P-loop NTPase superfamily) (42). These three motifs include the (i) Walker A/P-loop motif, (ii) sensor threonine (also known as the switch I motif), and (iii) Walker B/switch II motif. The Walker A motif is required for ATP binding and hydrolysis, while the sensor threonine and Walker B motifs are required for ATP hydrolysis because they help coordinate the Mg^2+^ ion cofactor. SpoIVA homologs lack the fourth motif, which confers specificity for GTP over ATP (42), consistent with the finding that SpoIVA hydrolyzes ATP instead of GTP (35).

Mutations of the three strictly conserved motifs in B. subtilis SpoIVA indicate that they are critical for SpoIVA function during sporulation (34, 35). Changing the conserved lysine in the Walker A motif to glutamate (K30E) prevented ATP binding *in vitro* and decreased B. subtilis heat-resistant spore formation by 10^8^-fold. However, if the lysine was changed to an alanine (K30A), heat-resistant spore formation decreased only 20-fold relative to the wild type (35). The relatively mild defect of a K30A mutation could result from the trace amount of ATP binding observed with SpoIVA_K30A_, which binds ATP ∼20-fold less efficiently than wild-type SpoIVA (35). Mutation of the strictly conserved sensor threonine to alanine (T70A) and its neighboring threonine (T71A) decreased heat-resistant spore formation by ∼10^4^ (34) (the dual mutations overcome the ability of Thr71 to partially substitute for the T70A mutation). Finally, mutation of the conserved aspartate (Asp97) in the SpoIVA Walker B motif reduced heat-resistant spore formation in B. subtilis by ∼10^7^ (34). Notably, sensor threonine and Walker B mutants retain the ability to bind ATP but cannot hydrolyze it (34).

Since all three of these NTPase motifs are conserved in C. difficile SpoIVA, we tested whether the Walker A, sensor threonine, and Walker B motifs were critical for C. difficile SpoIVA function during spore formation. We constructed mutations analogous to those generated in B. subtilis in these three motifs (34, 35). We mutated lysine 35 in the Walker A motif to either alanine or glutamate (K35A or K35E), sensor threonine 75 to alanine (T75A), and aspartate 102 in the Walker B motif to alanine (D102A). Since C. difficile SpoIVA does not carry a second threonine next to the sensor threonine, only a single threonine point mutation was made. The mutant alleles were introduced into either (i) the ectopic *pyrE* locus using *spoIVA* complementation constructs (43) or (ii) the native *spoIVA* locus using allelic exchange. We then determined the impact of these mutations on heat-resistant spore formation using a heat resistance assay. This assay measures the ratio of spores relative to total cells in sporulating cultures based on the ability of spores to survive heat treatment and outgrow to form colonies when plated onto media containing germinant (44). As a result, decreases in the heat resistance ratio can be caused by defects in spore formation, heat resistance, germination, and/or outgrowth.

**FIG 1:**
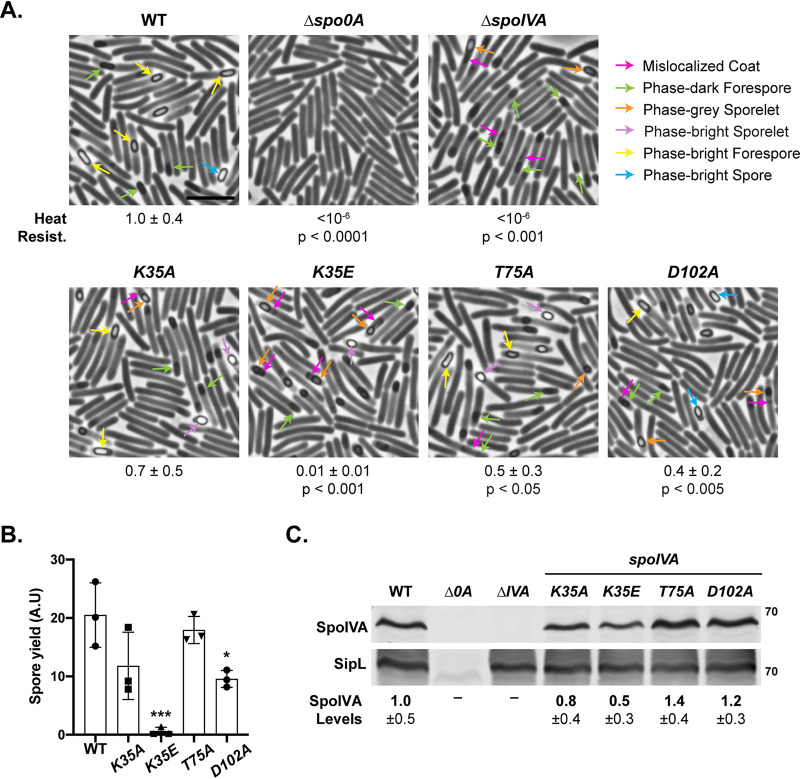
Effect of SpoIVA ATPase motif mutations encoded in native locus on functional spore formation and spore purification efficiency. (A) Phase-contrast microscopy analyses of the indicated C. difficile strains ∼20 h after sporulation induction. The SpoIVA ATPase motif mutations are encoded in the native *spoIVA* locus. Arrows mark examples of sporulating cells at different stages of maturation: pink arrows mark regions of the mislocalized coat based on previous studies (21, 25); green arrows highlight immature phase-dark forespores; orange arrows highlight phase-gray sporelets, which look swollen and are surrounded by a phase-dark ring; purple arrows highlight phase-bright sporelets, which are swollen and surrounded by a phase-dark ring; yellow arrows mark mature phase-bright forespores (phase brightness reflects cortex formation [39, 57]); and blue arrows highlight phase-bright free spores. Heat resistance efficiencies are based on 20- to 24-h sporulating cultures and represent the mean and standard deviation for a given strain relative to wild type based on a minimum of three biological replicates. The scale bar represents 5 μm. The limit of detection of the assay is 10^−6^. (B) Spore yields based on purifications from the indicated strains from three biological replicates. Yields were determined by measuring the optical density of spore purifications at 600 nm; yields are expressed in arbitrary units. (C) Western blot analyses of SpoIVA and SipL. SpoIVA levels were quantified based on analyses of three biological replicates using the procedure in reference 56. Statistical significance for all assays was determined relative to wild type using a one-way ANOVA and Tukey’s test. No statistically significant differences were detected for the Western blotting data. *, *P* < 0.05 ***, *P* < 0.005.

Strains encoding alanine substitutions in the Walker A, sensor threonine, and Walker B motifs (K35A, T75A, and D102A) resulted in only ∼3-fold reductions in heat-resistant spore formation relative to the wild type, regardless of whether the mutant *spoIVA* alleles were expressed from their native locus (Fig. 1) or the *pyrE* locus (see Fig. S2 in the supplemental material). Mutation of the Walker A lysine to glutamate (K35E) led to more-severe defects in heat-resistant spore formation relative to the wild type (∼100-fold defect), regardless of the chromosomal location of the *K35E* allele (Fig. 1A and Fig. S1A). The phenotype of the C. difficile *K35A* mutant was ∼7-fold less severe than an equivalent mutant in B. subtilis (35), while the phenotypes of the sensor threonine and Walker B mutants in C. difficile were at least 4,000-fold less severe than their counterparts in B. subtilis (34). While the heat resistance defect for *K35E* mutant was ∼10^2^-fold lower than wild-type C. difficile (Fig. 1A and Fig. S1), the equivalent mutation in B. subtilis led to a 10^8^-fold decrease in heat resistance (35). Thus, mutations in conserved NTPase motifs in C. difficile SpoIVA have relatively minor effects relative to the equivalent mutations in B. subtilis.

To determine the extent to which the ATPase motif mutations affected the number of spores produced, we measured the efficiency with which spores from the ATPase motif mutant strains could be purified. Spores were harvested from sporulating cultures grown on equal numbers of plates and purified using a Histodenz gradient (Fig. 1B). While the purification yield for the *T75A* mutant spores did not change relative to the wild type, the purification yields for *K35A* and *D102A* mutant spores decreased by ∼2-fold, although only the decrease for *D102A* mutant spores was statistically significant (*P* < 0.05). The purification yield for *K35E* mutant spore was ∼50-fold (*P* < 0.001) that of the wild type.

Phase-bright spores resembling wild-type spores were purified from the ATPase motif mutants carrying alanine mutations, although circular spores and spores with appendages were more frequently observed in the mutant strains relative to the wild type (see Fig. S3 in the supplemental material). The small amount of *K35E* spores that could be purified were primarily phase-bright sporelets, which are smaller and more swollen than wild-type spores (25, 40). Taken together, our results suggest that the heat resistance defect of the *K35E* mutant is mainly caused by a defect in spore assembly.

### ATPase motif mutations do not strongly affect SpoIVA levels in sporulating cells.

Since we previously found that mutating the Walker A lysine to glutamate reduced SpoIVA_K35E_ levels when recombinantly produced in E. coli (37), we measured the levels of SpoIVA and its binding partner SipL in the C. difficile ATPase motif mutant strains using Western blotting. These analyses revealed that SpoIVA levels were slightly but consistently reduced (∼2-fold) in the *K35E* mutant, although the difference was not statistically significant (Fig. 1C). The decrease in SpoIVA_K35E_ suggests that the K35E point mutation partially destabilizes SpoIVA in C. difficile, which is consistent with analyses in E. coli (37). However, it is unclear whether the ∼2-fold decrease in SpoIVA_K35E_ protein levels contributes significantly to the ∼100-fold heat resistance defect of the *K35E* mutant.

### Mutation of SpoIVA ATPase motifs leads to morphological defects in spore formation.

Although the effects of mutating the C. difficile SpoIVA ATPase motifs to alanine were relatively minor in the heat resistance assay, visual inspection of the purified spores (Fig. S3) and sporulating cells (Fig. 1 and Fig. S2) by phase-contrast microscopy revealed that the mutant spores had morphological defects relative to wild type. K35A, T75A, and D102A mutants often produced phase-bright sporelets, which are brighter and more swollen than the oblong forespore typically observed in the wild type (18). The alanine mutants also appeared to have a mislocalized coat associated with the forespore, since phase-gray areas appeared to extend like a “beard” from the forespore, a phenotype we previously observed in *spoVM* mutant cells (25). The *K35E* mutant produced phase-dark forespores, phase-dark sporelets, and phase-bright sporelets. Mislocalized coat was also observed associated with *K35E* mutant forespores, with the beards appearing to extend further into the mother cell cytosol of the *K35E* mutant and the parental Δ*spoIVA* strain (pink arrows, Fig. 1A; Fig. S2A).

**FIG 2:**
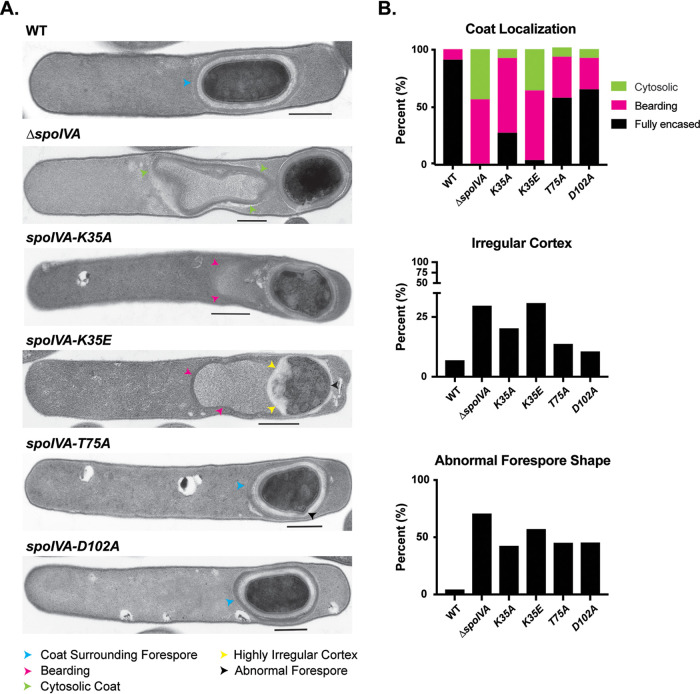
Coat and cortex abnormalities in *spoIVA* ATPase motif mutants. (A) Transmission electron microscopy (TEM) analyses of wild-type, Δ*spoIVA*, and *spoIVA* mutants carrying ATPase motif mutations in their native locus 23 h after sporulation induction. Scale bars represent 500 nm. Blue arrowheads mark properly localized coat, i.e., surrounding the entire forespore (FS); yellow arrowheads mark where the coat appears to detach from the forespore but stays partially associated, also known as “bearding” (25); and green arrowheads mark where the coat has fully detached from the forespore and is entirely cytosolic. A forespore with a cortex layer that varies markedly in thickness is highlighted with a yellow arrowhead. A misshapen forespore is marked with a black arrowhead. (B) Frequency of various phenotypes. At least 50 cells for each strain with visible signs of sporulation from a single biological replicate were analyzed.

To visualize the mislocalized coat with greater resolution, we analyzed the ATPase motif mutants using transmission electron microscopy (TEM). Analyses of ∼50 sporulating cells that had completed engulfment revealed that Walker A motif mutants frequently exhibited coat encasement defects, with the coat encasing the forespore in only ∼20% of *K35A* mutant spores and ∼5% of *K35E* mutant spores (Fig. 2). The coat encasement defect of the *K35E* mutant was almost as severe as Δ*spoIVA*, for which no coat encasement was observed.

The Walker A mutations resulted in the coat appearing to slough off the forespore at a frequency similar to that of the parental Δ*spoIVA* sporulating cells (50% to 60%) (Fig. 2). We previously termed this phenotype “bearding” (25). The *K35E* mutation also increased the frequency of the coat completely mislocalizing to the cytosol similar to the Δ*spoIVA* mutant (∼35% to 40% of cells). Coat mislocalization defects were comparatively less severe in the sensor threonine and Walker B *T75A* and *D102A* mutants, with most of these mutants completing coat encasement. Nevertheless, bearding was still observed in ∼20% to 30% of these mutant cells (Fig. 2).

In addition to the coat localization defects, SpoIVA ATPase motif mutants were more likely to produce cortex layers of highly irregular thickness than wild-type cells. Over 30% of *K35E* and Δ*spoIVA* sporulating cells produced highly irregular cortex layers (Fig. 2), while this defect was observed in only ∼5% of wild-type cells. Irregular cortex was also observed in 20% of *K35A* and ∼10% of *T75A* and *D102A* mutants. These cortex abnormalities were frequently associated with changes in forespore shape. Notably, almost half of the alanine ATPase motif mutants and even more of the *K35E* and Δ*spoIVA* mutants produced forespores that were irregularly shaped, whether they were more circular in shape as seen by phase-contrast microscopy (Fig. 1), exhibited protrusions, or appeared wavy (Fig. 2). Since we have reported a similar defect in cortex morphology in *spoVM* mutants (25), SpoVM and SpoIVA would appear to modulate cortex synthesis on an unknown level in C. difficile.

### ATPase motif mutations decrease SpoIVA encasement of the forespore.

We next sought to determine the effects of the ATPase motif mutations on SpoIVA localization around the forespore because B. subtilis mutants lacking ATPase activity fail to fully encase the forespore (34, 35). In B. subtilis, disrupting ATP binding by mutating the Walker A lysine to alanine (K30A) caused SpoIVA to localize to a single cap at the mother cell proximal pole, while mutation of the lysine to glutamate (K30E) redistributed SpoIVA to the cytosol (35). Disrupting ATP hydrolysis (but not ATP binding) due to mutations of the Walker B motif in B. subtilis still allowed SpoIVA to localize to both poles of the forespore but prevented full encasement of the forespore in >80% of cells (34).

To assess whether the C. difficile ATPase motif mutations decreased the ability of SpoIVA to encase the forespore, we introduced *mCherry-spoIVA* alleles carrying the ATPase motif mutations into the ectopic *pyrE* locus of strains carrying the same *spoIVA* mutant allele in their native locus (e.g., *K35A*/*mCherry-spoIVA_K35A_*). These strains carry an untagged SpoIVA ATPase motif mutant along with an mCherry-SpoIVA ATPase motif mutant because wild-type mCherry-SpoIVA is not fully functional unless it is coproduced with untagged wild-type SpoIVA (25).

**FIG 3:**
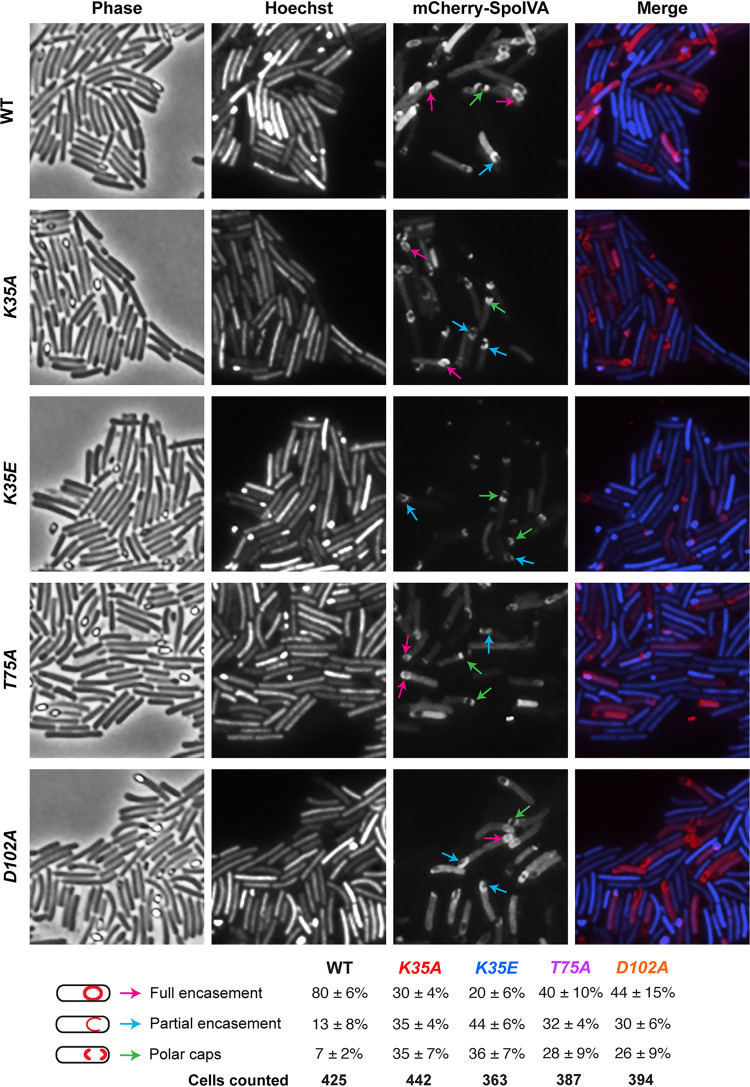
Effect of SpoIVA ATPase motif mutations on SpoIVA localization. Fluorescence microscopy analyses of wild type and *spoIVA* mutants encoding ATPase motif mutations in their native locus 23 h after sporulation induction. Phase-contrast microscopy was used to visualize sporulating cells (phase). Hoechst staining used to visualize the nucleoid is shown in blue, and mCherry-SpoIVA fluorescence is shown in red. The merged images of the Hoechst staining and mCherry signal are shown. Schematic of coat localization phenotypes quantified is shown along with the percentage of cells in a given strain that exhibited this phenotype. The average percentages and standard deviations shown are based on counts from three biological replicates, with multiple images from each replicate being quantified. The total numbers of cells counted are also shown.

While wild-type mCherry-SpoIVA localized primarily around the forespore in ∼80% of cells ∼24 h after sporulation was induced, mutation of the ATPase motifs reduced mCherry-SpoIVA encasement of the forespore (Fig. 3). The sensor threonine and Walker B mutants encased the forespore at an ∼2-fold lower frequency than that of the wild type, with T75A and D102A mCherry-IVA either partially encasing the forespore or “capping” both poles of the forespore. The localization defects were slightly more severe in the *K35A* Walker A motif mutant than in the T75A and D102A alanine mutants. In contrast, the K35E mutation resulted in mCherry-SpoIVA mainly exhibiting a capped distribution (either single or double capped) and surrounding the forespore in only ∼20% of cells.

The K35E mutation noticeably diminished the intensity of mCherry-IVA signal relative to wild type and the other ATPase motif mutants, but this was not due to mCherry being liberated from mCherry-SpoIVA_K35E_ through proteolysis (see Fig. S4 in the supplemental material). However, less mCherry-IVA_K35E_ was observed by Western blotting, which is consistent with Western blot analyses of the untagged SpoIVA variant (Fig. 1). Taken together, our results suggest that mutating C. difficile SpoIVA ATPase motifs decreases forespore encasement by SpoIVA but not as severely as in B. subtilis (34, 35).

### Coat protein encasement of the forespore is reduced in SpoIVA ATPase motif mutants.

**FIG 4:**
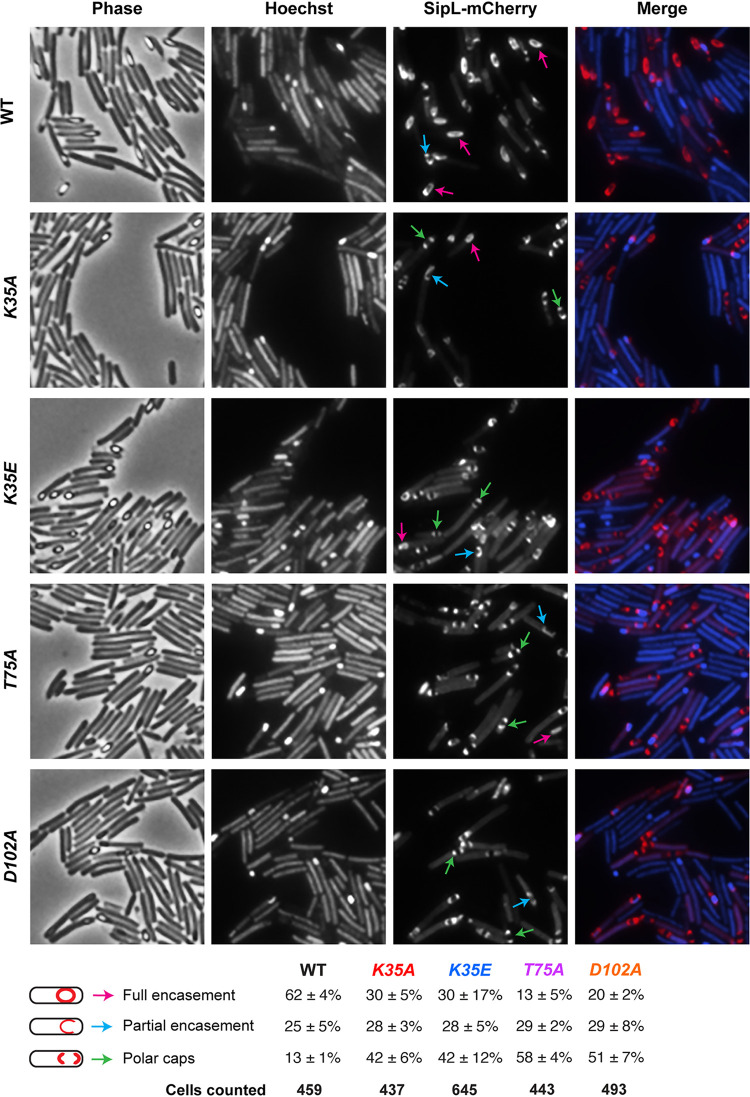
Effect of SpoIVA ATPase motif mutations on SipL localization. Fluorescence microscopy analyses of Δ*sipL* complemented with *sipL-mCherry* (designated “WT”) and *spoIVA* mutants encoding ATPase motif mutations in their native locus from which *sipL* was deleted and *sipL-mCherry* was integrated into the *pyrE* locus. Microscopy was performed on samples 23 h after sporulation induction. Phase-contrast microscopy was used to visualize sporulating cells (phase). Hoechst staining used to visualize the nucleoid is shown in blue, and SipL-mCherry fluorescence is shown in red. The merged images of the Hoechst staining and mCherry signal are shown. Schematic of coat localization phenotypes quantified is shown along with the percentage of cells in a given strain that exhibited this phenotype. The average percentages and standard deviations shown are based on counts from three biological replicates, with multiple images from each replicate being quantified. The total number of cells counted is also shown.

C. difficile SpoIVA is required to recruit SipL to the forespore (40), so we tested whether the ATPase motif mutations decreased SipL localization using a SipL-mCherry fluorescent protein fusion we previously constructed (40). Since this fusion protein encases the forespore more efficiently when SipL-mCherry is the only form of SipL present in the cell (40), we deleted the *sipL* gene from the *spoIVA* mutants encoding ATPase motif mutations in their native loci. We then integrated a construct encoding SipL-mCherry into the *pyrE* locus. While SipL-mCherry localized to the forespore in all the ATPase motif mutants tested, SipL-mCherry encased the forespore ∼2- to 3-fold less frequently in these mutants than in the wild type (Fig. 4). Indeed, in most (>70%) SpoIVA ATPase motif mutant cells, SipL-mCherry was detected capping either both poles of the forespore or the pole closest to the mother cell.

To test whether the SpoIVA ATPase motif mutations affected the localization of coat proteins found in the outer layers of the spore as suggested by the coat bearding observed by TEM (Fig. 2), we analyzed the localization of mCherry fusion to the spore surface protein the CotE mucinase (45). To localize C. difficile CotE, we introduced a construct encoding a CotE-mCherry fusion protein (18) into the *pyrE* locus of the ATPase motif mutant strains. In wild-type cells, C. difficile CotE encases the forespore in a typically nonuniform fashion, concentrating at the forespore poles as two caps (18, 21). In the absence of SpoIVA, CotE-mCherry failed to localize to the forespore and instead formed a bright focus in the cytosol of sporulating cells (see Fig. S5 in the supplemental material), as seen with prior studies of a CotE-SNAP fusion protein in a *spoIVA* mutant (21). However, when the SpoIVA ATPase motifs were mutated to alanine, CotE-mCherry still localized to both poles of the forespore (Fig. S5), although CotE-mCherry formed a single bright focus close to the forespore of the *K35E* Walker A mutant (rather than two polar caps) more frequently than the other mutant strains. Unfortunately, the nonuniform distributions of CotE-mCherry around the forespore made it difficult to quantify the different localization patterns observed. Regardless, relative to a *spoIVA* null mutant, the C. difficile ATPase motif mutations did not severely disrupt the localization of CotE-mCherry.

### Mutation of the SpoIVA Walker A motif decreases SpoIVA binding to SipL.

**FIG 5:**
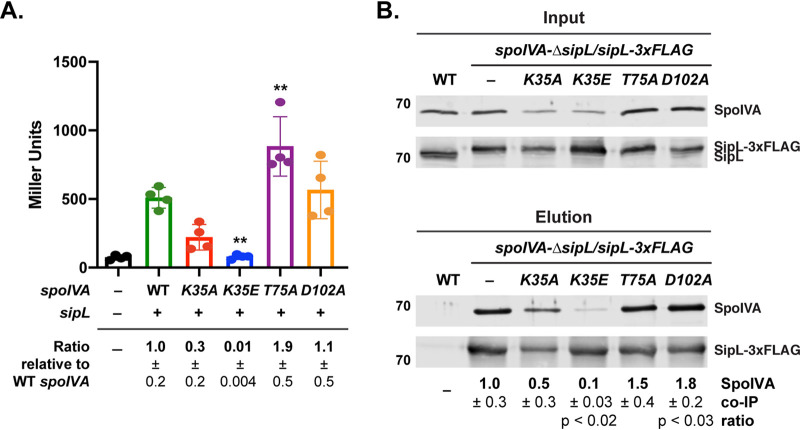
Binding of SipL to SpoIVA ATPase motif mutants. (A) Bacterial adenylate cyclase two-hybrid (BACTH) analysis of SipL binding to SpoIVA ATPase motif mutants. Plasmids encoding pKNT25-*sipL* and pUT18C-*spoIVA* ATPase motif variants were cotransformed into E. coli BTH101. After the transformation plates were incubated at 30°C for 40 h, cells were scraped off the plate, and β-galactosidase activity (shown in Miller units) was measured to quantify the interaction between SipL and the SpoIVA variants. Assays were performed in technical triplicate for three biological replicates. – refers to the empty vector. Statistical significance for all assays was determined relative to wild type using a one-way ANOVA and Tukey’s test. **, *P* < 0.01. (B) Coimmunoprecipitation analysis of SpoIVA ATPase motif mutants binding to FLAG-tagged SipL. SipL-3VMBF-0029-2015 FLAG was immunoprecipitated from cleared lysates (“input” fraction) prepared from either wild type (WT), the Δ*sipL/sipL-3×FLAG* complementation strain (–), or Δ*sipL/sipL-3×FLAG* strains encoding SpoIVA ATPase motif mutations in their native locus. Proteins bound to anti-FLAG magnetic beads were eluted using FLAG peptide (“elution” fraction). Wild type served as a negative control for the anti-FLAG beads. Lysates were prepared from strains induced to sporulate for 22 h. The immunoprecipitations shown are representative of three biological replicates. The pulldown efficiency shown is the amount of SpoIVA that was pulled down relative to SipL. The efficiency of pulldown was determined by calculating amount of SpoIVA that coimmunoprecipitated with SipL as a bait. This ratio was set to 1 for WT and then the relative pulldown efficiencies for the SpoIVA ATPase mutants were determined. Ratios represent the mean and standard deviation for a given strain relative to wild-type SpoIVA based on three biological replicates.

Our localization analyses with SipL-mCherry revealed that SipL localizes to the forespore but encases it less efficiently when the SpoIVA ATPase motifs are mutated (Fig. 4). Given that a loss of SpoIVA causes SipL-mCherry to redistribute to the cytosol (40), our data suggest that the SpoIVA ATPase motif mutants retain the ability to bind and recruit SipL to the forespore. However, prior coaffinity purification analyses with SpoIVA and SipL in E. coli indicated that the SpoIVA K35E strongly impairs binding to SipL (37). To address this potential discrepancy, we quantified the interaction between SpoIVA and SipL using (i) a bacterial two-hybrid assay in E. coli and (ii) coimmunoprecipitation analyses in C. difficile (Fig. 5).

When an adenylate cyclase-based bacterial two-hybrid assay (46) was used to measure SpoIVA ATPase motif mutant binding to SipL, we observed that the Walker A mutations reduced SpoIVA binding to SipL by ∼3-fold for SpoIVA_K35A_ (*P* = 0.06) and ∼100-fold for SpoIVA_K35E_ binding (*P* < 0.003) (Fig. 5a). The latter result mirrors those previously obtained with prior coaffinity purification analyses (37), although it is also possible that the reduced binding we observed in the two-hybrid analyses is exacerbated by decreases in the stability of the K35A and K35E SpoIVA variants relative to wild-type (WT) SpoIVA. Surprisingly, the sensor threonine mutation increased SpoIVA binding to SipL by 2-fold relative to WT SpoIVA (*T75A*, *P* < 0.01), while the Walker B mutation did not significantly affect SpoIVA binding to SipL (Fig. 5a).

While the two-hybrid assay in E. coli allowed us to quantify the direct interaction between SpoIVA and SipL, this assay takes place in the absence of other C. difficile sporulation proteins and outside the context of the forespore. Since localization of B. subtilis SpoVM and SpoIVA to the forespore cooperatively enhances their encasement of the forespore by increasing their local concentrations (33), we considered the possibility that SpoIVA-SipL binding is stabilized in the context of the forespore. To determine how the SpoIVA ATPase motif mutations affect binding to SipL during sporulation, we immunoprecipitated FLAG-tagged SipL from a previously constructed strain that expresses *sipL-3×FLAG* from the *pyrE* locus of Δ*sipL* (40). We then measured the amount of untagged SpoIVA ATPase motif mutant variants that were also pulled down. To perform these analyses, we used our double *spoIVA*-Δ*sipL* mutant strains where *sipL* has been deleted from strains carrying SpoIVA ATPase motif mutations in the native *spoIVA* locus. We then complemented this double-mutant strain with a construct encoding SipL-3×FLAG integrated into the *pyrE* locus such that the only copy of SipL made is FLAG tagged.

Immunoprecipitation of SipL-3×FLAG from these strains revealed that mutations of the Walker A, but not sensor threonine or Walker B, motif reduced the amount of SpoIVA that coimmunoprecipitated with SipL-3×FLAG. Approximately 3-fold less SpoIVA_K35A_ and 10-fold less SpoIVA_K35E_ (*P* < 0.02) relative to wild-type SpoIVA coimmunoprecipitated with SipL-3×FLAG. While slightly less SpoIVA_K35E_ was observed in the input fraction, unbound SpoIVA_K35E_ was observed in the flow-through fraction, indicating that SpoIVA_K35E_ levels were not limiting in the coimmunoprecipitation analyses (see Fig. S6 in the supplemental material). The Walker B mutations resulted in more SpoIVA being pulled down with SipL-3×FLAG, with 1.5-fold more SpoIVA_T75A_ and 1.8-fold more SpoIVA_D102A_ (*P* < 0.03) being coimmunoprecipitated. Taken together, the two hybrid analyses in E. coli and coimmunoprecipitation analyses in sporulating C. difficile cells indicate that mutations in the Walker A motif decrease C. difficile SpoIVA binding to SipL. They further suggest that SpoIVA sensor threonine and Walker B mutations enhance SipL binding. Given that sensor threonine and Walker B mutations have been shown to “trap” B. subtilis SpoIVA in an ATP-bound conformation (34), our results imply that SipL preferentially recognizes the ATP-bound form of SpoIVA.

### Synergistic effects of combining SipL and SpoIVA Walker A mutations on functional spore formation.

Unfortunately, we could not directly test this hypothesis because C. difficile SpoIVA is largely insoluble in E. coli unless coproduced with SipL (37), making it difficult to purify sufficient quantities for biochemical analyses. Given that C. difficile SpoIVA shares 71% homology with B. subtilis SpoIVA, it is likely that the Walker A motif mutations disrupt C. difficile SpoIVA ATP binding, whereas the sensor threonine or Walker B mutations do not. Nevertheless, to examine this hypothesis further, we reasoned that combining the SpoIVA K35A Walker A motif mutation with SipL mutations that reduce binding to SpoIVA might strongly impair the interaction between SpoIVA and SipL binding and exacerbate the relatively minor heat resistance defect (∼3-fold) of the *spoIVA K35A* mutant (Fig. 1). In contrast, combining the SpoIVA sensor threonine or Walker B motif mutations with the same SipL mutations would not be expected to affect heat-resistant spore formation as strongly because the SpoIVA variants presumably retain binding to ATP and thus bind SipL at levels equal or greater to wild-type SpoIVA (Fig. 5).

We previously identified two SipL mutations that reduce SipL-SpoIVA binding in coimmunoprecipitation analyses and SipL function in heat resistance assays, namely, I463R and W475E (40). The I463R mutation strongly impaired SpoIVA binding to SipL but surprisingly decreased functional spore formation only by ∼10-fold, while the W475E mutation partially reduced SpoIVA binding to SipL but decreased functional spore formation by ∼100-fold (40). Since the W475E mutation appeared to affect SipL function beyond its ability to disrupt binding to SpoIVA, we reasoned that combining the *spoIVA K35A* Walker A allele with the *sipL W475E* allele might impair functional spore formation less severely than combining the *spoIVA* Walker A mutant allele with the *sipL I463R* allele, since the latter allele reduces binding to SpoIVA more severely than the *sipL W475E* allele.

**FIG 6:**
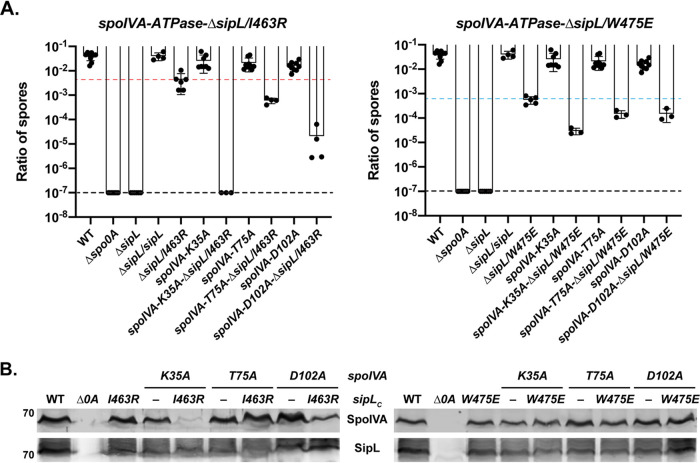
Allele-specific effects of combining SipL and SpoIVA mutations on functional spore formation. (A) The indicated C. difficile strains were induced to sporulate for 20 to 24 h. The SpoIVA ATPase motif mutations are carried in the native *spoIVA* locus, while the SipL LysM domain mutations are carried in a *sipL* gene integrated into the *pyrE* locus of a Δ*sipL* strain. The ratio of heat-resistant spores to total CFU was determined from a minimum of three biological replicates. The ratio shown is the mean and standard deviation for a given strain relative to wild type. The limit of detection of the assay is 10^−6^ (black line). The red and blue lines indicate the spore ratio for the *sipL I463R* and *W475E* complementation strains, respectively, which had the most severe phenotype of the *sipL* and *spoIVA* single point mutations. Statistical significance for all assays was determined relative to the wild type using a one-way ANOVA and Tukey’s test. (B) Western blot analyses of SpoIVA and SipL.

To generate the double point-mutant strains, we complemented the *spoIVA*-Δ*sipL* ATPase motif double mutants used in Fig. 4 with *sipL* complementation constructs encoding either the I463R or W475E mutations. We analyzed only the alanine mutations of the SpoIVA ATPase motifs because the relatively minor heat resistance defects of these mutants (2- to 3-fold) (Fig. 1) would allow us to detect synergistic effects more readily. Combining the *sipL I463R* allele with the *spoIVA K35A* Walker A mutant allele exacerbated the heat resistance defects of the individual mutations so severely that no heat-resistant spores were detected in the *spoIVA_K35A_ sipL_I463R_* double-mutant strain (Fig. 6A). This represents a 10^4^-fold decrease in heat resistance relative to the *sipL I463R* single mutant, whose heat resistance defect is slightly more severe than the *spoIVA K35A* single mutant. Consistent with the severe heat resistance defect of the *spoIVA_K35A_ sipL_I463R_* double mutant, its sporulating cells resembled those of the *sipL* deletion strain (see Fig. S7 in the supplemental material). In contrast, combining the *sipL I463R* allele with the *spoIVA T75A* sensor threonine (*spoIVA_K35A_ sipL_I463R_*) resulted in only a 50-fold decrease in heat-resistant spore formation, which is only 6-fold more severe than the single *sipL I463R* mutant. Combining the *sipL I463R* allele with the *spoIVA D102A* Walker A mutant allele resulted in a 1,000-fold decrease in heat resistance for the double mutant; this defect was ∼60-fold more severe than the *sipL I463R* single mutant. Interestingly, the severity of the *sipL_I463R_ spoIVA* ATPase alanine motif double mutants mirrored the binding (affinities) observed in the bacterial two-hybrid assay (Fig. 5A) and the SpoIVA levels in these mutants (Fig. 6B). SpoIVA_K35A_ was barely detectable in the *spoIVA_K35A_ sipL_I463R_* double mutant even though its levels were only slightly decreased in the *spoIVA_K35A_* single mutant (Fig. 6B).

Importantly, when the SpoIVA ATPase motif alanine mutations were combined with the *sipL W475E* allele, the heat resistance defects of the double mutants were largely indistinguishable from each other, and no change in SpoIVA levels was observed in the double-mutant strains relative to wild-type or the single-mutant strains. Since the deleterious effect of combining the Walker A K35A mutation with the SipL I463R mutation appears to be allele specific, our results are consistent with the hypothesis that SipL specifically recognizes the ATP-bound form of SpoIVA and strongly suggest that Ile463 of SipL plays a critical role in recognizing this form of SpoIVA.

## DISCUSSION

While B. subtilis and C. difficile use different pathways for localizing coat proteins to the forespore (11, 37, 47), recent work has shown that these organisms can exhibit differential requirements for conserved proteins. In this study, we identify another differential requirement for a conserved spore morphogenetic protein between B. subtilis and C. difficile. Even though both organisms require the conserved coat morphogenetic protein SpoIVA to recruit coat proteins to the forespore and make functional spores (27, 36, 37, 40), we show here that the predicted ATPase activity of SpoIVA is largely dispensable for heat-resistant spore assembly in C. difficile, in contrast with B. subtilis. While alanine mutations that specifically disrupt SpoIVA ATP hydrolysis in B. subtilis (i.e., sensor threonine and Walker B mutations) result in severe (>10^4^-fold) decreases in functional spore formation (34), we determined that the analogous mutations in C. difficile SpoIVA result in only 2- to 3-fold decreases in heat-resistant spore formation (Fig. 1; see Fig. S1 in the supplemental material) and minimally disrupt coat localization to the forespore (Fig. 2 and Fig. S5). Even the most severe ATPase motif mutation we identified, *K35E*, decreased functional C. difficile spore formation by only 100-fold (Fig. 1), whereas the equivalent mutation reduced spore formation by 10^8^-fold in B. subtilis (35).

The differential requirement for the SpoIVA ATPase motifs between B. subtilis and C. difficile likely reflects the absence of a quality control mechanism for removing spores with defects in encasing SpoIVA around the forespore in C. difficile. In B. subtilis, this mechanism removes spores with mutations in *spoVM* or *spoIVA*, including SpoIVA ATPase mutants (29), by inducing lysis of the mother cell through the action of a small, *Bacillales*-specific protein (39), CmpA. The B. subtilis quality control mechanism has been proposed to prevent mutations that generate spores of inferior quality from overtaking a population (29, 38). Since C. difficile *spoIVA* ATPase motif mutants and *spoVM* mutants both exhibit coat and cortex abnormalities (Fig. 2 and 4 and Fig. S5) (25), C. difficile would appear to tolerate spores with morphological abnormalities more readily than B. subtilis. Given that resistant spore formation is essential for C. difficile to survive outside the host and during passage through the stomach (7), the infection cycle presumably places sufficient selective pressure on C. difficile to prevent mutants with even minor spore assembly defects from overtaking the population. It would be interesting to test this model by analyzing the effect of the ATPase motif mutations on infectious dose and transmission over many cycles in an animal model of infection, although such analyses would be quite labor intensive and costly.

The differential requirement may also reflect functional redundancy in the C. difficile spore assembly process such that a loss of SpoIVA ATPase activity could be compensated by other morphogenetic factors. In B. subtilis, SpoVM and SpoIVA form a mutually dependent ratchet that drives assembly of the coat on the forespore surface (33). SpoVM helps recruit SpoIVA to the positively curved surface of the forespore, while local polymerization of SpoIVA on top of SpoVM enhances SpoVM binding to the forespore membrane (33). Since our data imply that C. difficile SipL preferentially binds SpoIVA in its ATP-bound conformation, it is possible that reduced binding of SpoIVA Walker A mutants to SipL is compensated by SpoIVA Walker A mutant binding to SpoVM. For example, the *K35E* C. difficile SpoIVA Walker A mutation decreases binding to SipL by 100-fold in a heterologous bacterium but only 10-fold in coimmunoprecipitation analyses performed in sporulating C. difficile (Fig. 5A). Despite this reduced binding, mCherry-SpoIVA_K35E_ still encases the forespore in 20% of *spoIVA K35E* cells, and SipL-mCherry also encases the forespore of ∼30% of *spoIVA K35E* cells (Fig. 3). These results suggest that, despite the predicted inability of C. difficile SpoIVA_K35E_ to polymerize, other factors (potentially SpoVM) appear to allow C. difficile SpoIVA_K35E_ to associate with and encase the forespore even though SpoIVA_K35E_ binds SipL much less efficiently (Fig. 5). Constructing a *spoVM-spoIVA K35E* double mutant would provide insight into this question as would detailed analyses of purified SpoVM, SpoIVA, and SipL binding to membranes, similar to the work performed with purified B. subtilis SpoVM and SpoIVA by Peluso et al. (33).

Consistent with the hypothesis that redundant mechanisms stabilize C. difficile SpoIVA ATPase motif mutant binding to and encasement of the forespore, C. difficile *spoIVA* ATPase motif alanine mutants fully encased the forespore in ∼30% of Walker A *K35A* mutant cells and ∼40% of sensor threonine *T75A* and Walker B *D102A* mutants (Fig. 3). In contrast, the equivalent B. subtilis Walker A mutation completely abrogated SpoIVA_K35A_ encasement of the forespore, with SpoIVA_K35A_ forming only a single cap on the mother cell proximal forespore membrane (35), and sensor threonine and Walker B mutations resulted in only ∼15% of B. subtilis fully encasing the forespore (34). While the forespore encasement defects of B. subtilis SpoIVA ATPase mutants are consistent with their inability to polymerize into SpoIVA filaments (34, 35), lateral interactions between polymerization-defective SpoIVA proteins may allow mutant SpoIVA to partially encase the forespore (33). These lateral interactions may form more readily with C. difficile SpoIVA irrespective of its putative ATPase activity compared with B. subtilis SpoIVA given the relatively high percentage of C. difficile *spoIVA* ATPase motif mutant cells that complete forespore encasement. An important goal of future work is to determine whether C. difficile SpoIVA binds ATP and hydrolyzes it, similar to its B. subtilis homolog, for which it shares 50% identity and 69% similarity.

While biochemical analyses of C. difficile SpoIVA are challenging because of its low solubility when produced in the absence of SipL in E. coli (37), future work should also assess whether C. difficile SpoIVA undergoes conformational rearrangements upon binding ATP and then hydrolyze ATP similar to those reported for B. subtilis SpoIVA (34, 41). Determining how C. difficile SpoIVA binding to SipL affects these conformational rearrangements will provide important insight into how SpoIVA and SipL collectively regulate spore coat assembly. In addition, crystallographic analyses comparing C. difficile SpoIVA alone with SpoIVA bound to SipL would address this question as well as the role of SipL Ile463 in potentially recognizing the ATP-bound form of SpoIVA based on our analyses. Regardless, given the importance of spores to C. difficile infection, understanding the mechanisms underlying SpoIVA and SipL function during sporulation could aid in the development of antisporulation therapies, which were recently shown to prevent disease recurrence in an animal model of infection (48).

## MATERIALS AND METHODS

### Bacterial strains and growth conditions.

The C. difficile strains used are listed in Table S1 in the supplemental material. All strains derive from the erythromycin-sensitive 630Δ*ermΔpyrE* parental strain, which is the sequenced clinical isolate 630, which we used for *pyrE*-based allele-coupled exchange (ACE) (43). Strains were grown on brain heart infusion supplemented (BHIS) with yeast extract and cysteine (49), taurocholate (TA; 0.1% [wt/vol]; 1.9 mM), cefoxitin (8 μg/ml), and kanamycin (50 μg/ml) as needed. For ACE, the C. difficile-defined medium (50) (CDDM) was supplemented with 5-fluoroorotic acid (5-FOA; at 2 mg/ml) and uracil (at 5 μg/ml).

The Escherichia coli strains used for HB101/pRK24-based conjugations and bacterial adenylate cyclase two-hybrid (BACTH) assay plasmid preparations are listed in Table S2 in the supplemental material. E. coli strains were grown at 37°C with shaking at 225 rpm in Luria-Bertani (LB) broth. The medium was supplemented with ampicillin (50 μg/ml), chloramphenicol (20 μg/ml), or kanamycin (30 μg/ml) as needed.

### E. coli strain construction.

All primers used for cloning are listed in Table S2. Details of E. coli strain construction are provided in the Text S1 in the supplemental material. All plasmid constructs were sequence confirmed using Genewiz and transformed into DH5α. The HB101/pRK24 E. coli strain was used to conjugate sequence-confirmed plasmids into C. difficile.

### C. difficile strain construction and complementation.

Allele-coupled exchange (ACE) (43) was used to introduce the *spoIVA* ATPase motif point mutations back into the native locus of a parental Δ*spoIVA* Δ*pyrE* strain. Using this strain facilitated colony PCR screening to identify strains that had restored the native *spoIVA* locus with the mutant gene supplied. ACE was also used to introduce the *sipL* deletion using strain number 1704 pMTL-YN3 Δ*sipL* into 630Δ*erm*Δ*pyrE spoIVA* ATPase mutants. Complementations were performed as previously described by conjugating HB101/pRK24-carrying pMTL-YN1C plasmids into Δ*pyrE*-based strains (51) using ACE.

### Plate-based sporulation assay.

C. difficile strains were grown overnight from glycerol stocks on BHIS plates supplemented with TA (0.1% [wt/vol]). Colonies from these plates were inoculated into BHIS liquid medium and back diluted 1:25 once they were in stationary phase (∼3 h later). The cultures were grown until they reached an optical density at 600 nm (OD_600_) between 0.4 and 0.7; 120 μl was used to inoculate 70:30 agar plates (37) where sporulation was induced for 20 to 24 h. Cells were analyzed by phase-contrast microscopy and harvested for Western blot analysis.

### Heat resistance assay on sporulating cells.

Heat-resistant spore formation was measured in C. difficile sporulating cultures between 20 and 24 hours as previously described (44). Heat resistance efficiencies represent the average ratio of heat-resistant CFU obtained from functional spores in the sample for a given strain relative to the average ratio determined for the wild type based on a minimum of three biological replicates. The ratio of functional, heat-resistant spores shown for a given strain represents the average of every assay performed for the manuscript. Statistical significance was determined using one-way analysis of variance (ANOVA) and Tukey’s test.

### Western blot analyses.

Samples for Western blot analyses were prepared as previously described (37). Briefly, sporulating cell pellets were resuspended in 100 μl of phosphate-buffered saline (PBS), and 50-μl samples were freeze-thawed for three cycles and then resuspended in 100 μl of EBB buffer (8 M urea, 2 M thiourea, 4% [wt/vol] SDS, 2% [vol/vol] β-mercaptoethanol). The samples were boiled for 20 min, pelleted at high speed, and resuspended in the same buffer to maximize protein solubilization. Finally, the samples were boiled for 5 min and pelleted again at high speed. Samples were resolved on 12% SDS-PAGE gels and then transferred to an Immobilon-FL polyvinylidene difluoride (PVDF) membrane, where they were blocked in Odyssey blocking buffer with 0.1% (vol/vol) Tween 20. Rabbit anti-SipL_ΔLysM_ and mouse anti-SpoIVA (52) antibodies were used at a 1:2,500 dilution. Rabbit anti-mCherry (Abcam, Inc.) was used at a 1:2,000 dilution. IRDye 680CW and 800CW infrared dye-conjugated secondary antibodies were used at a 1:20,000 dilution, and blots were imaged on an Odyssey LiCor CLx imaging system.

### Spore purification.

Sporulation was induced in 70:30 agar plates for 2 to 3 days as previously described (25, 53). C. difficile sporulating cells were washed 5 to 6 times in ice-cold water, incubated overnight at 4°C, and treated with DNase I (New England BioLabs) at 37°C for 60 minutes. Finally, spores were purified on a HistoDenz gradient (Sigma-Aldrich) and resuspended in water; spore purity was determined by phase-contrast microscopy (>95%), and the optical density of the spore preparation was measured at OD_600_. Spore yields were quantified by measuring the OD_600_ of the spore purifications from four 70:30 plates per replicate. The average of three biological replicates was calculated, and statistical significance was determined using a one-way ANOVA and Tukey’s test. Spores were stored in water at 4°C.

### TEM analysis.

Sporulating cultures (23 to 24 h) were fixed and processed for electron microscopy by the University of Vermont Microscopy Center as previously described (37). A minimum of 50 full-length sporulating cells were used for phenotype counting.

### mCherry fluorescence microscopy.

Live-cell fluorescence microscopy was performed using Hoechst 33342 (15 μg/ml; Molecular Probes) and mCherry protein fusions. Samples were prepared on agarose pads either as previously described (21) or agarose pads prepared on Gene Frames (ThermoScientific). Briefly, one gene frame sticker was placed on a microscope slide, and 350 μl of 1% agarose was deposited inside the frame. Another microscope slide was set on top of the frame containing the agarose. The agarose pad was allowed to cool for 10 minutes at 4°C and then dried at room temperature for 5 minutes. Sporulating cultures were pipetted onto the agarose pad, covered with a cover glass, and imaged. Images were taken 30 minutes after harvesting the C. difficile sporulating cultures to allow reconstitution of the mCherry fluorescence signal in an obligate anaerobe.

Phase-contrast and fluorescence microscopy was carried out on a Nikon 60× oil immersion objective (1.4 numerical aperture [NA]) using a Nikon 90i epifluorescence microscope. A CoolSnap HQ camera (Photometrics) was used to acquire multiple fields for each sample in 12-bit format with 2-by-2 binning, using NIS-Elements software (Nikon). The Texas Red channel was used to acquire images after 300 ms for mCherry-SpoIVA, 90 ms for SipL-mCherry, and 400 ms for CotE-mCherry. Hoechst stain was visualized using a 90-ms exposure time, and 3-ms exposure was used for phase-contrast pictures. Finally, 10-MHz images were imported to Adobe Photoshop CC 2017 software for pseudocoloring and minimal adjustments in brightness and contrast levels. Protein localization analysis were performed on a minimum of three independent biological replicates.

### Immunoprecipitation analyses.

Immunoprecipitations with strains producing SipL-3×FLAG were performed on lysates prepared from cultures induced to sporulate on 70:30 plates for 24 h. The samples were processed as previously described (40), except that anti-FLAG magnetic beads (Sigma-Aldrich) were used to pull down FLAG-tagged proteins and any associated proteins. All immunoprecipitations were performed on three independent biological replicates.

### Bacterial two-hybrid analyses.

Bacterial adenylate cyclase two-hybrid (BACTH) assays were performed using E. coli BTH101 cells based on the system first described by Karimova et al. (46). Briefly, for each assay, BTH101 cells were freshly cotransformed with ∼100 ng of each BACTH assay plasmid. The transformations were incubated on LB agar supplemented with 50 μg/ml kanamycin, 50 μg/ml carbenicillin, and 0.5 mM isopropyl-β-d-thiogalactopyranoside (IPTG) for 40 h at 30°C. After this incubation period, β-galactosidase activity was quantified in Miller units using the protocol described in references 54 and 55).

### Quantification of Western blots.

Western blots from three biological replicates were quantified using the sum of all data points method (56) as previously described (52). The signal for SpoIVA, SipL, and FLAG antibodies was set to zero for the negative-control strains, i.e., Δ*spoIVA*, Δ*sipL*, and Δ*sipL/sipL* strains, respectively. For the coimmunoprecipitation quantifications, the amount of SpoIVA and SipL was measured in the elution fraction by quantifying the bands detected by Western blotting. The pulldown efficiency was determined by comparing the SpoIVA levels relative to SipL levels based on the quantification described above. The resulting SpoIVA/SipL ratio was then set to 1 for the WT strain and the relative amounts of SpoIVA to SipL coimmunoprecipitation and the efficiency of pulldown relative to WT were determined for the ATPase mutants.

## Supplementary Material

Supplemental file 1
